# Supplementary material for: SpotClean adjusts for spot swapping in spatial transcriptomics data
Source: Nat Commun. 2022 May 27;13:2971. doi: 10.1038/s41467-022-30587-y (PMC9142522; doi:10.1038/s41467-022-30587-y)
Supplement: Supplementary file 2 — Supplementary Information [file 41467_2022_30587_MOESM2_ESM.pdf]

Supplement to "SpotClean adjusts for spot swapping in spatial transcriptomics data"

Zijian Ni[1], Aman Prasad[2], Shuyang Chen[1], Richard B. Halberg[3,4], Lisa M. Arkin[2], Beth A. Drolet[2], Michael A. Newton[1,5], Christina Kendziorski[5]

These authors contributed equally: Zijian Ni, Aman Prasad

Corresponding author: Christina Kendziorski (kendzior@biostat.wisc.edu)

[1]Department of Statistics, University of Wisconsin-Madison, Madison, WI, USA

[2]Department of Dermatology, University of Wisconsin-Madison, Madison, WI, USA

[3]Department of Medicine, University of Wisconsin-Madison, Madison, WI, USA

[4]Department of Oncology, University of Wisconsin-Madison, Madison, WI, USA

[5]Department of Biostatistics and Medical Informatics, University of Wisconsin-Madison, Madison, WI, USA

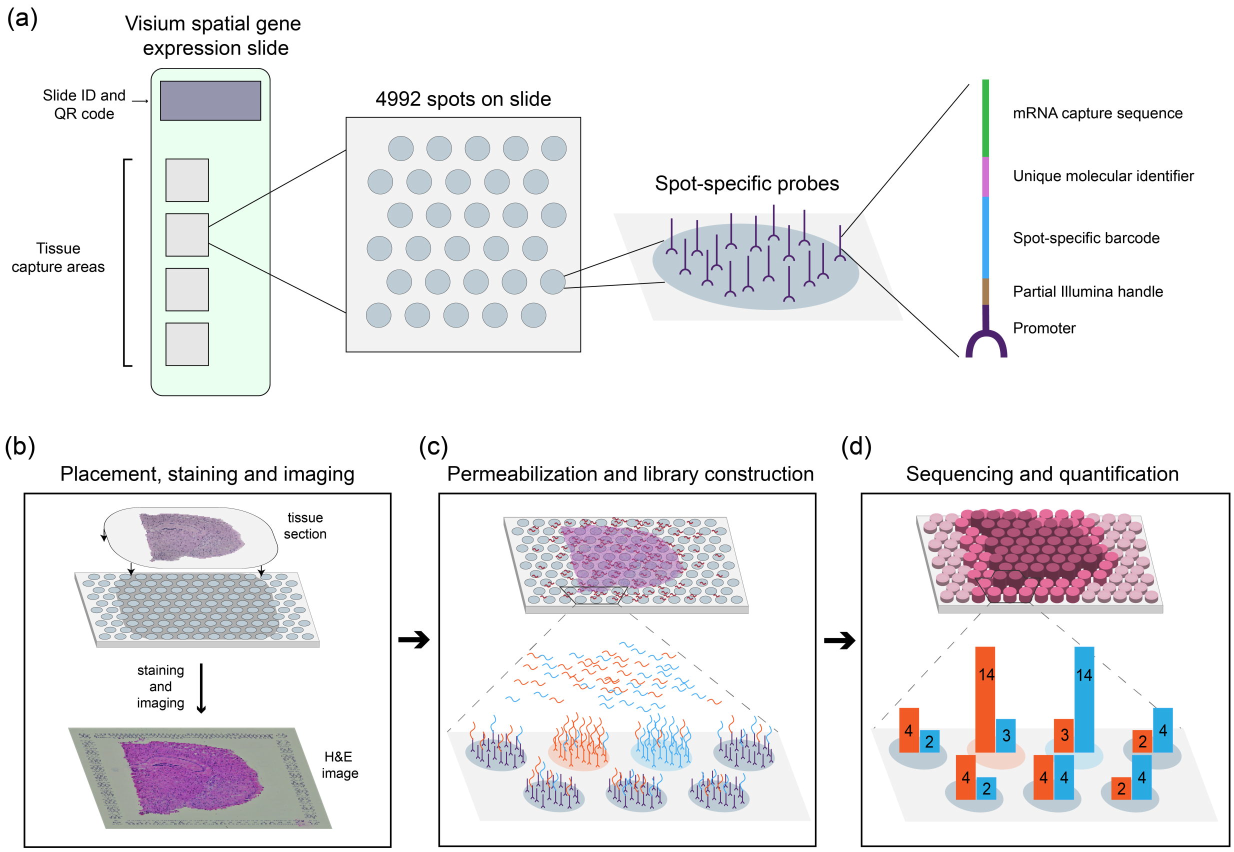

Supplementary Figure 1: Overview of the 10x Genomics Visium (10x)[1] spatial transcriptomics experiment. (a) The 10x Visium spatial gene expression slide contains four tissue capture sites for processing multiple tissue samples simultaneously. Each capture site contains 4992 spots, with each spot containing millions of spot-specific probes that bind mRNA. (b) Fresh frozen or FFPE tissue is sectioned, placed on a capture area, and imaged, typically via Hematoxylin and Eosin (H&E) staining. (c) Following imaging, the tissue is permeabilized to release mRNA. The lower panel shows five background spots (gray) and two tissue spots (orange and blue). Due to spot swapping, mRNAs from one tissue spot bind probes at other spots. (d) The bound mRNAs are released, processed, sequenced and quantified to give a gene-by-spot matrix of UMI counts. In this hypothetical example, due to spot swapping, UMI counts at each of the seven spots are a mixture of mRNAs from the two distinct tissue spots.

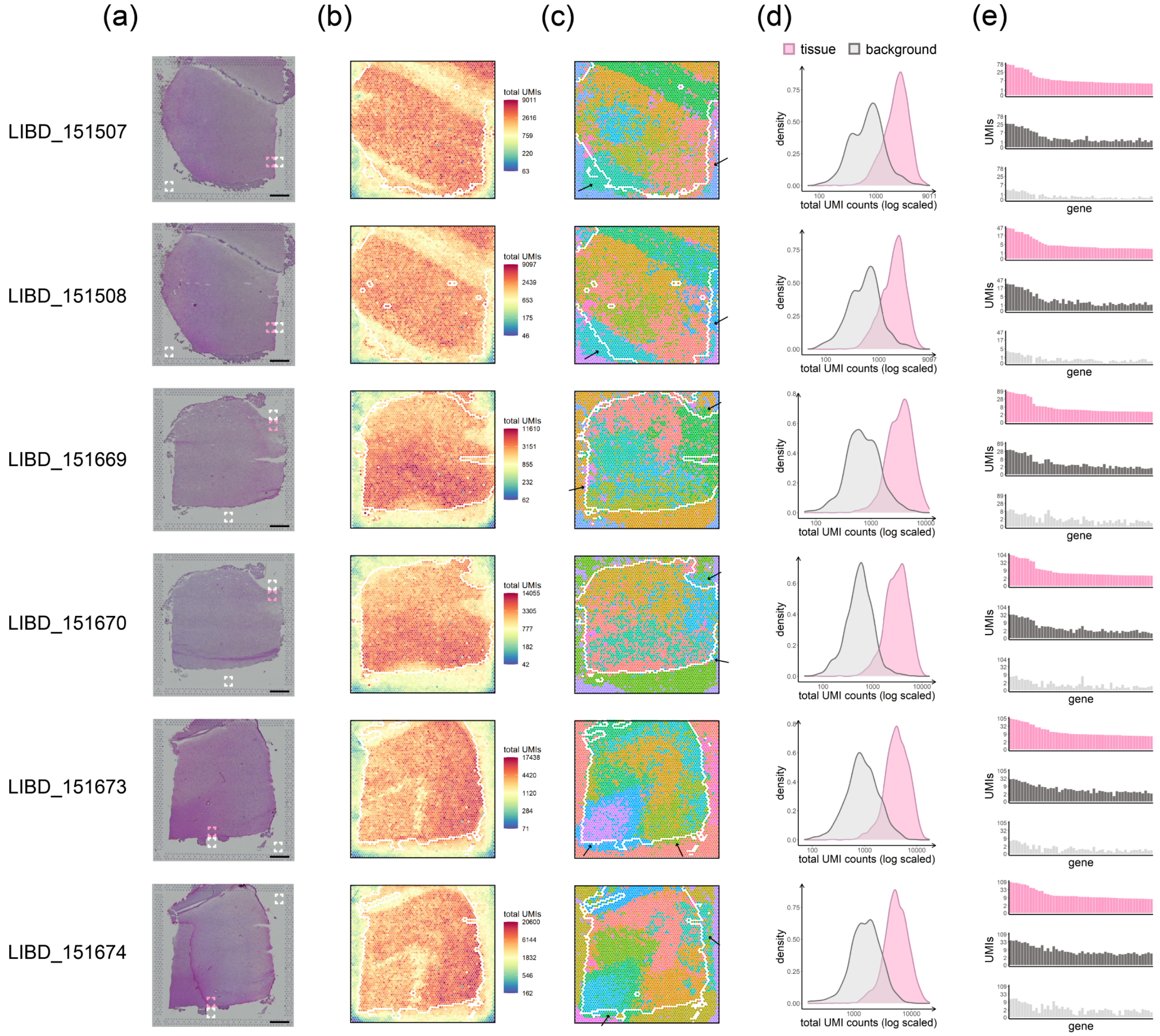

Supplementary Figure 2: Data from six human brain samples from Maynard *et al.*[2]. (a) H&E images for the six different samples. Scale bar is 1mm. (b) UMI total counts in the background decrease with increasing distance from the tissue. (c) Spots on the slide are colored by their cluster membership via graph-based clustering (clusters not shown). Black arrows highlight areas of spot swapping. (d) UMI count densities for tissue and background spots show relatively high counts in the background. (e) Counts of the top 50 genes (genes with highest total UMI expression) from a select tissue region (upper), from a nearby background region (middle), and from a distant background region (bottom) show the similarity between expression in tissue spots and nearby background spots due to spot swapping from tissue to background, an effect that decreases as distance from the tissue increases. The tissue region and background regions used for each sample are highlighted in panel (a) in pink and white, respectively. Tissue spots on the perimeter (shown in white in panels (b) and (c)) were removed prior to calculating the summaries in panels (d)-(e) in an effort to ensure that the effects shown are not due to spots on the tissue-background boundary.

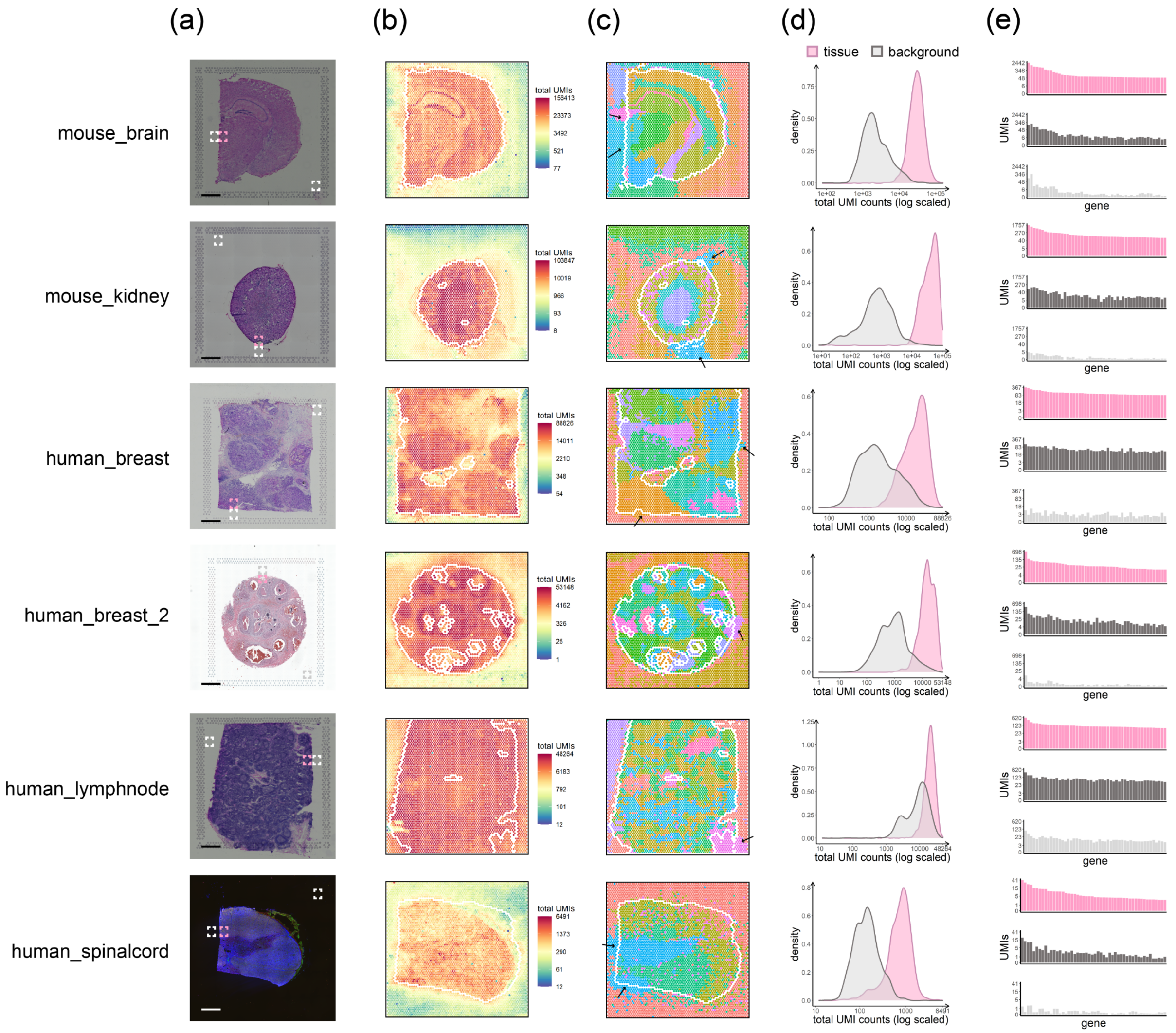

Supplementary Figure 3: Data from six publicly available 10x Genomics datasets. (a) H&E images for the six different samples. Scale bar is 1mm. (b) UMI total counts in the background decrease with increasing distance from the tissue. (c) Spots on the slide are colored by their cluster membership via graph-based clustering (clusters not shown). Black arrows highlight areas of spot swapping. (d) UMI count densities for tissue and background spots show relatively high counts in the background. (e) Counts of the top 50 genes (genes with highest total UMI expression) from a select tissue region (upper), from a nearby background region (middle), and from a distant background region (bottom) show the similarity between expression in tissue spots and nearby background spots due to spot swapping from tissue to background, an effect that decreases as distance from the tissue increases. The tissue region and background regions used for each sample are highlighted in panel (a) in pink and white, respectively. There is considerable overlap of tissue and background spots in the UMAP plots. Tissue spots on the perimeter (shown in white in panels (b) and (c)) were removed prior to calculating the summaries in panels (d)-(e) in an effort to ensure that the effects shown are not due to spots on the tissue-background boundary.

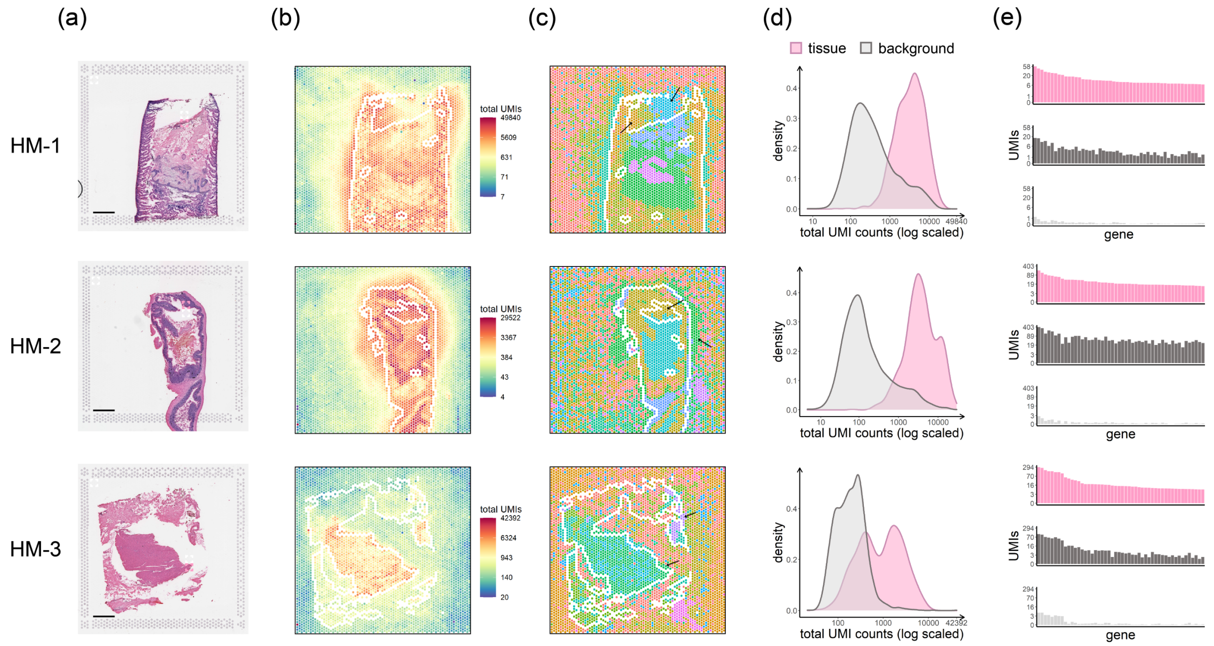

Supplementary Figure 4: Data from the three chimeric samples composed of human and mouse tissues. (a) H&E images for the three different samples. Scale bar is 1mm. (b) UMI total counts in the background decrease with increasing distance from the tissue. (c) Spots on the slide are colored by their cluster membership via graph-based clustering (clusters not shown). Black arrows highlight areas of spot swapping. (d) UMI count densities for tissue and background spots show relatively high counts in the background. (e) Counts of the top 50 genes (genes with highest total UMI expression) from a select tissue region (upper), from a nearby background region (middle), and from a distant background region (bottom) show the similarity between expression in tissue spots and nearby background spots due to spot swapping from tissue to background, an effect that decreases as distance from the tissue increases. The tissue region and background regions used for each sample are highlighted in panel (a) in pink and white, respectively. There is considerable overlap of tissue and background spots in the UMAP plots. Tissue spots on the perimeter (shown in white in panels (b) and (c)) were removed prior to calculating the summaries in panels (d)-(e) in an effort to ensure that the effects shown are not due to spots on the tissue-background boundary.

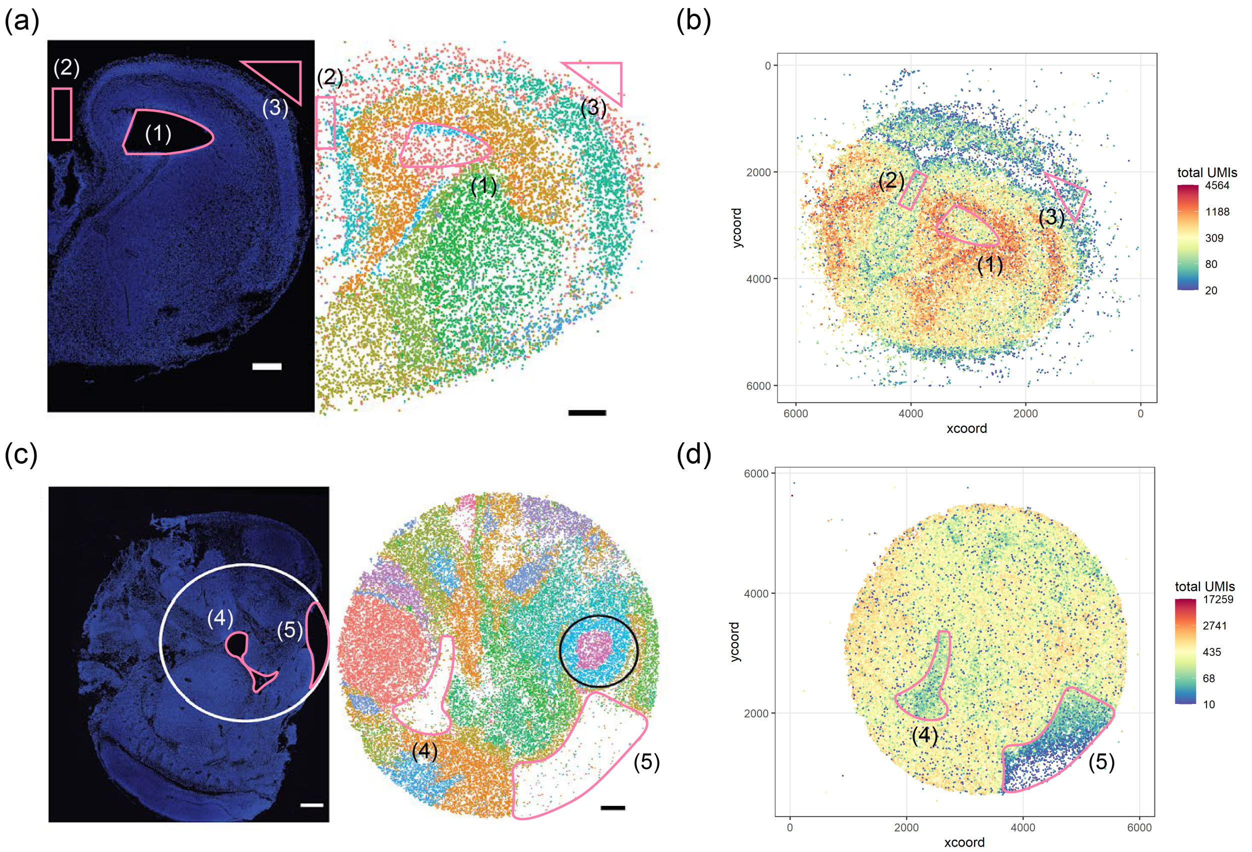

Supplementary Figure 5: Evidence of spot swapping in Slide-seqV2 data. (a) DAPI stain of E15 mouse brain (left) and Slide-seqV2 data of E15 brain with cluster labels (right) reported in Stickels *et al.*[3]. Three background regions identified from the DAPI image are shown in pink and labeled as (1), (2) and (3). The same regions are also identified in the graph-based clustering of beads. (b) Raw UMI counts data colored by total UMI counts for all spatial barcodes shows positive UMI counts detected in the three background regions. (c)-(d) are identical to (a)-(b), but for the E12.5 mouse embryo data. The black circle shown in (c) is part of the original image and not relevant here. Scale bar is 200μm in (a) and (c).

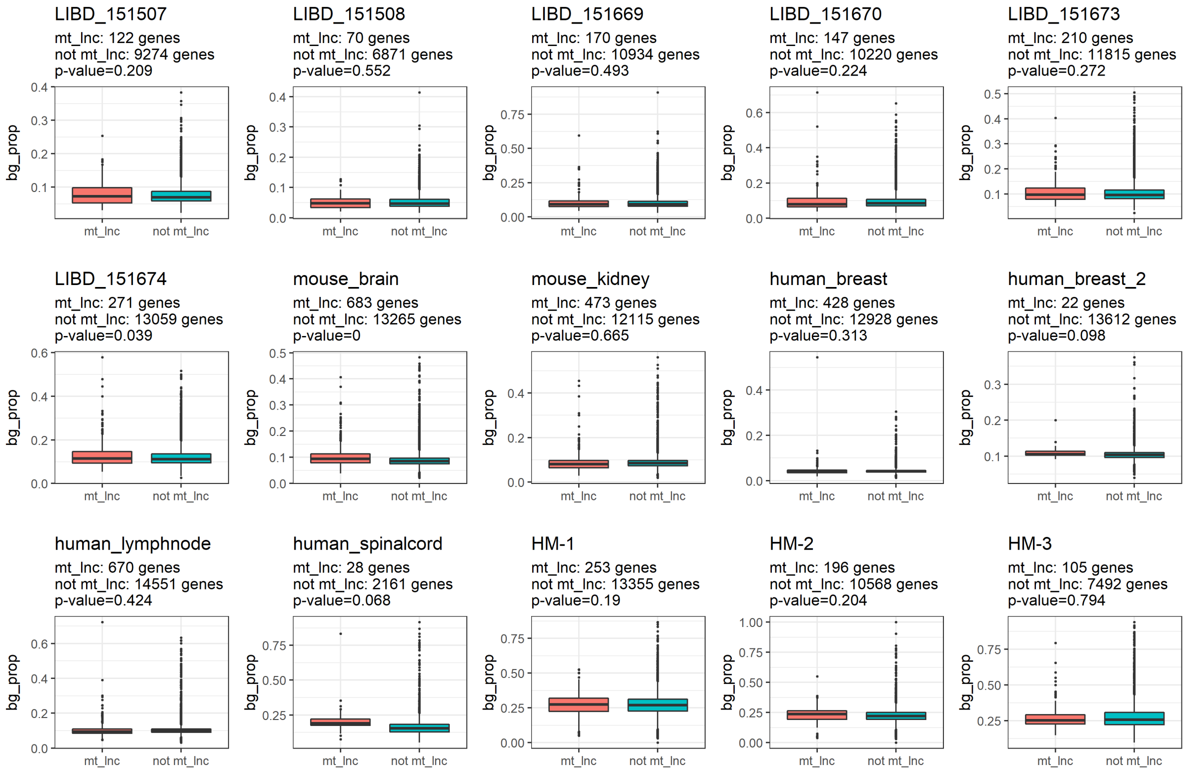

Supplementary Figure 6: Boxplots of proportion of UMI counts in background spots for mitochondrial genes and long non-coding RNAs (mt_lnc) and the remaining genes (not mt_lnc) in 12 publicly available datasets as well as the chimeric experiments. Two-sample two-sided t-tests were conducted to evaluate whether the differences between mt_lnc and not mt_lnc are significant. Two datasets (LIBD_151674 and mouse_brain) show significant differences at unadjusted p-value<=0.05 (and only mouse_brain if adjusting for multiple tests), where the estimated bleeding rates are higher in mitochondrial genes and long non-coding RNAs than the remaining genes. In general, there is no clear pattern where mitochondrial genes and long non-coding RNAs have higher or lower bleeding rates than the remaining genes. The number of genes in the boxplots are shown in panel subtitles. The lower whisker, lower hinge, line inside box, upper hinge, and upper whisker represent the minimum, lower quartile, median, upper quartile, and maximum calculated without outlier values which are more than 1.5 * inter-quartile range away from the hinges and are shown in separate dots.

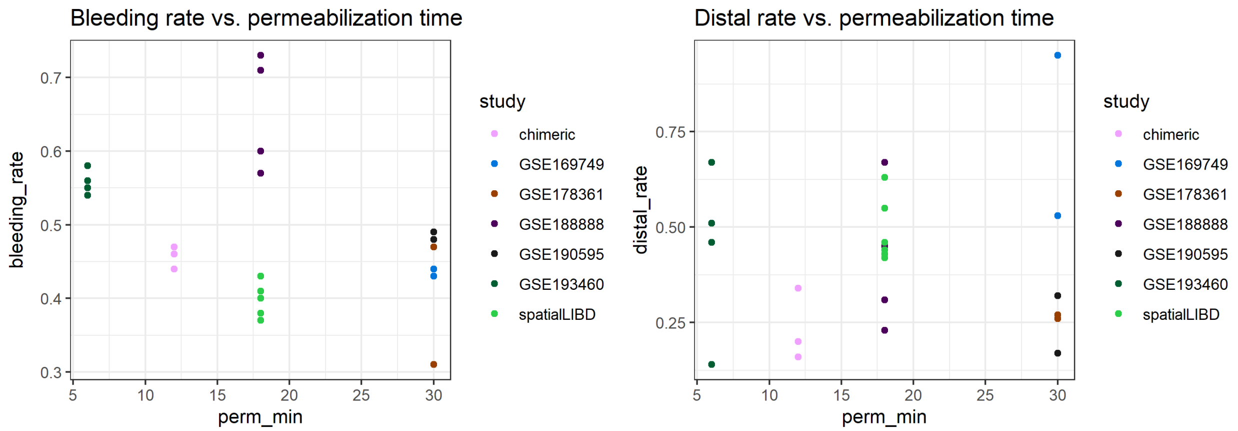

Supplementary Figure 7: Relationship between bleeding rate estimated via SpotClean and permeabilization time (left) and between distal contamination rate estimated via SpotClean and permeabilization time (right). No clear patterns were observed between estimated bleeding rates and permeabilization times, or between estimated distal contamination rates and permeabilization times.

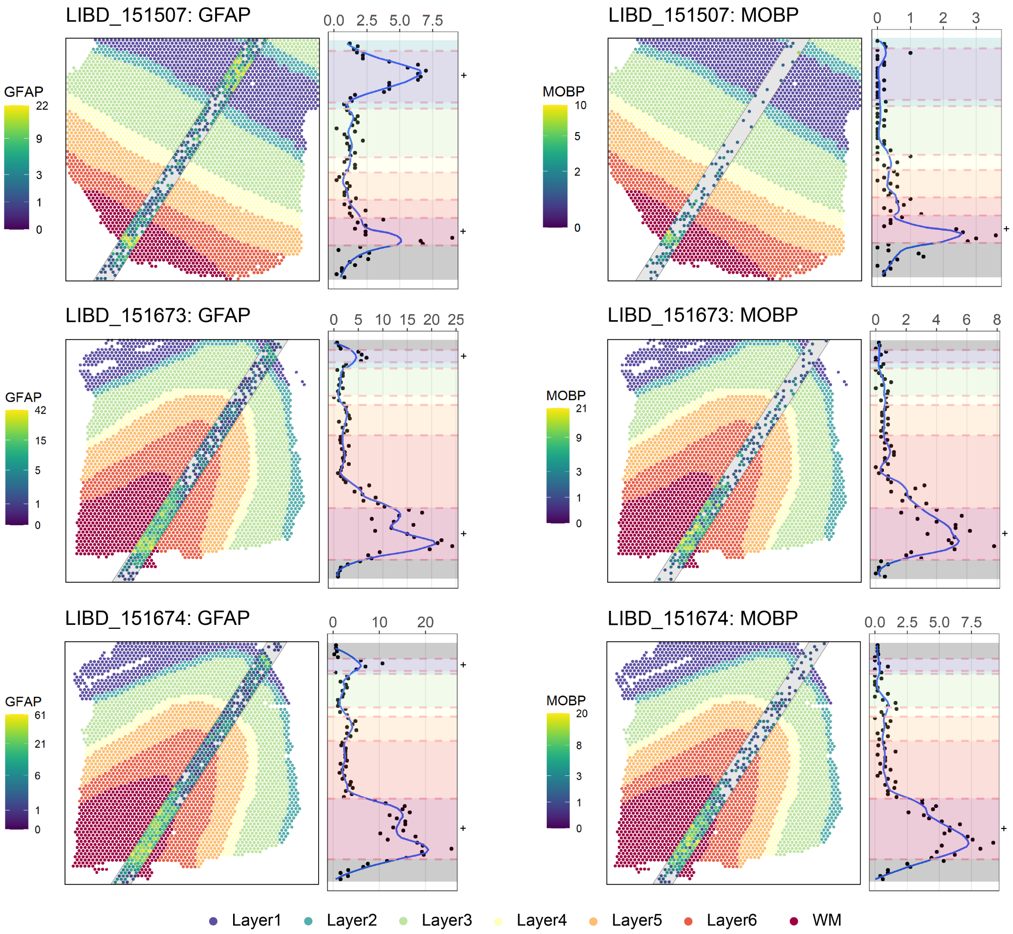

Supplementary Figure 8: The effect of spot swapping on layer-specific marker genes in three human brain sample datasets. The upper left panel shows the annotated LIBD_151507 sample and a stripe with width equal to five spots. Each spot in the stripe is colored by expression of GFAP, a marker for white matter (WM) and Layer1; the "+" denotes the regions where marker expression is expected to be high (here, WM and Layer 1). Average expression of each row in the stripe is shown in the right subpanel. The average is taken across the five spots for every row contained completely within a layer; for rows containing two layers, the average is taken across the three or four spots making up the major layer. When spot swapping occurs, marker expression is relatively high in nearby layers, as observed here. While it is possible that some increase in marker expression in adjacent tissue spots may be due to the presence of WM (or Layer1) cells at those spots, we note that the rate of expression decay into the background spots (where no cells are present) is similar to the rate of decay into adjacent tissue regions. Consequently, the possible presence of cells from a given layer in adjacent tissue spots outside that layer is not sufficient to fully explain the observed expression patterns shown here. The lower middle and lower left panels show the same plot for different tissue samples; the right panels show MOBP, another WM marker.

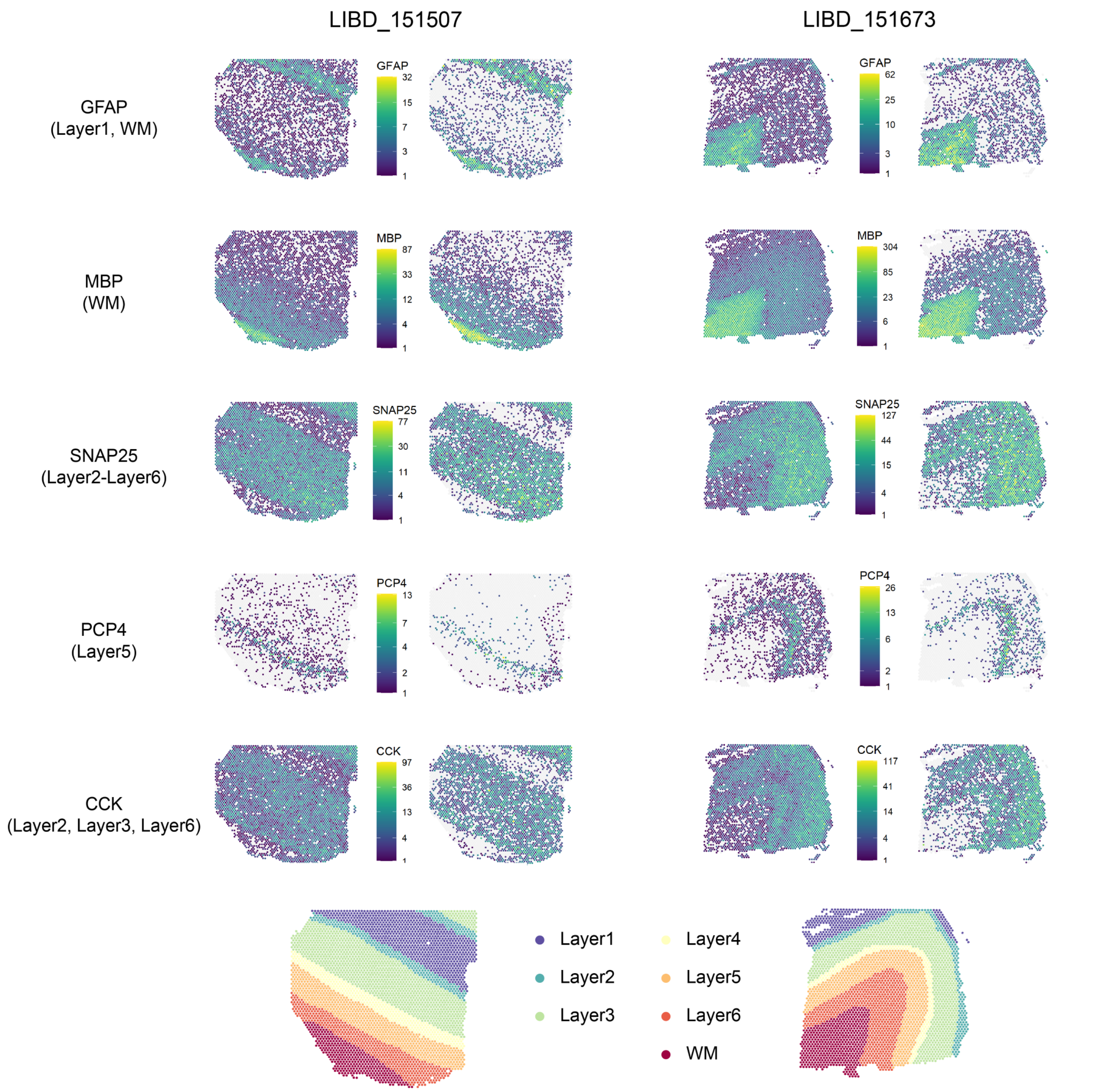

Supplementary Figure 9: Gene expression heatmaps for GFAP, MBP, SNAP25, PCP4, CCK in brain samples are shown for the raw and SpotClean decontaminated data. Columns 1 and 3 show raw expression in brain samples LIBD_151507 and LIBD_151673; columns 2 and 4 show SpotClean decontaminated data for these same samples. Brain layer annotations are also shown at the bottom. SpotClean maintains expression in the marker layers and reduces expression in adjacent layers, thereby increasing marker specificity.

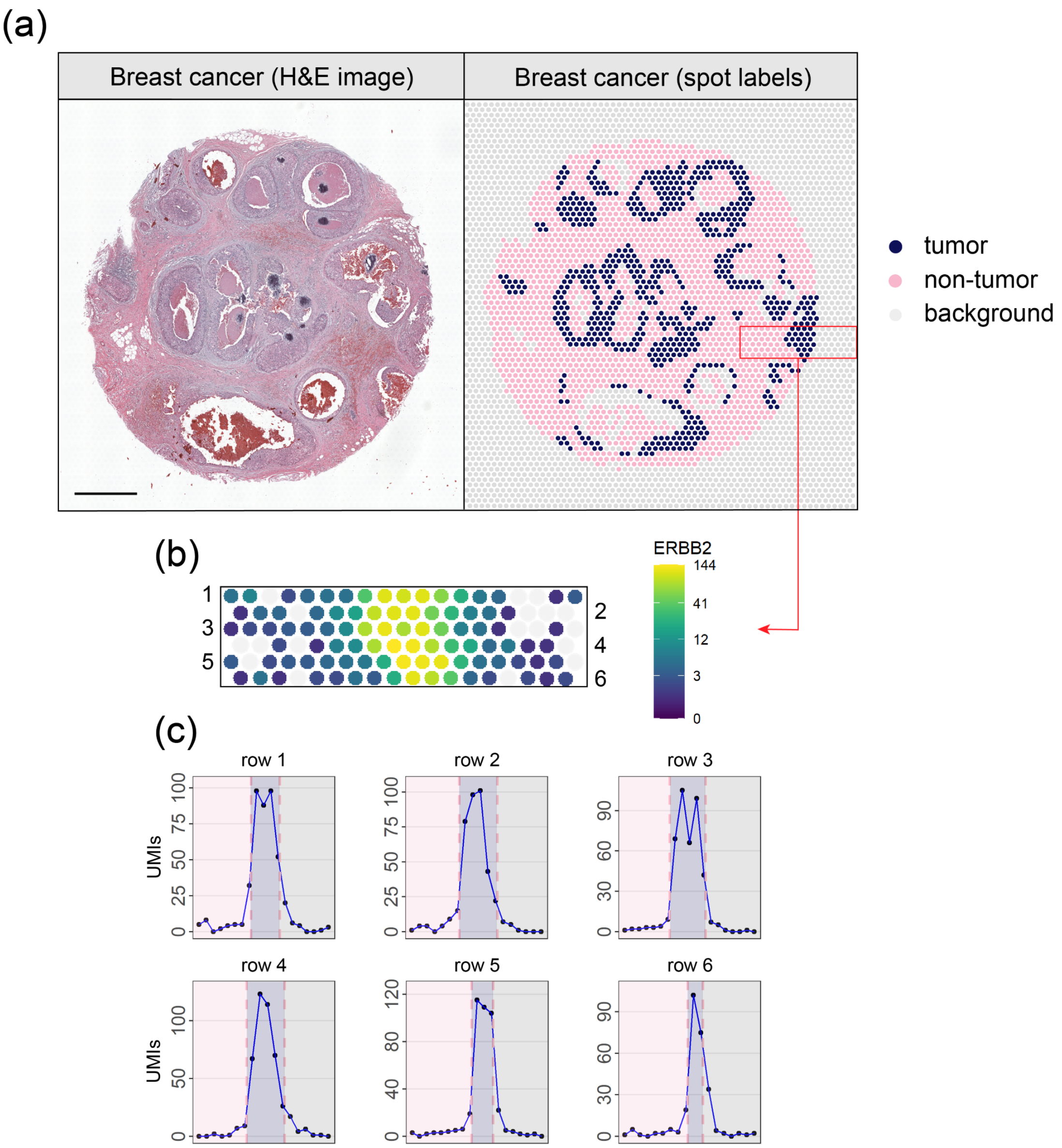

Supplementary Figure 10: The effect of spot swapping on breast cancer specific marker genes in a human breast cancer sample, human_breast_2. Panel (a) shows the H&E image (left) and spots annotated as tumor, non-tumor, and background via visual inspection (right). Scale bar is 1mm. Panel (b) shows high expression for ERBB2 in the tumor spots of the inset; also shown is relatively high expression in adjacent spots that decreases with increasing distance from the tumor. Panel (c) shows ERBB2 expression for spots in each row of the inset. Some of the decrease into adjacent spots shown in panels (b) and (c) may be due to the presence of both tumor and normal cells in spots near the tumor tissue. However, this is unlikely given that the rate of decay into the adjacent spots is similar to the rate of decay into the background (where no tissue is present).

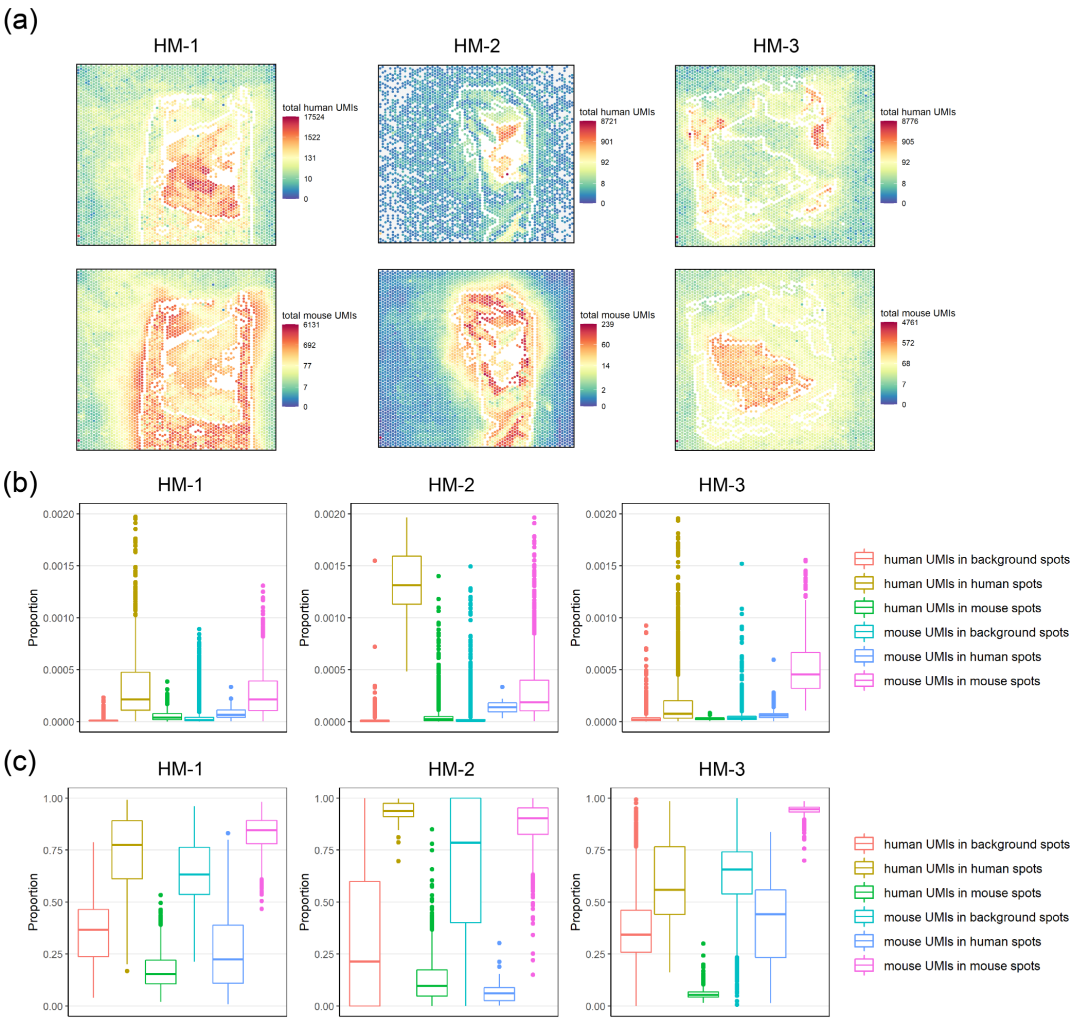

Supplementary Figure 11: Panel (a) shows total UMI counts in human-specific genes (upper) and mouse-specific genes (lower) for the three chimeric experiments. Panel (b) shows the proportion (out of total UMIs) of spot-swapped UMI counts (human-specific UMIs in background or mouse spots; mouse-specific UMIs in background or human spots). Also shown are the proportion of human-specific UMIs in human spots and mouse-specific UMIs in mouse spots. Note that there may be spot-swapped reads in these latter proportions (e.g. reads from human spot t bound by probes at human spot t'), but they cannot be identified in this experiment. Panel (c) shows spot-specific proportions. Tissue spots on the perimeter as well as spots annotated as mixtures (shown in white in panel (a)) were removed prior to calculating the summaries in panels (b) and (c) in an effort to ensure that the effects shown are not due to spots on the tissue-background boundary. For the six boxplots from left to right, n=2962, 751, 1014, 2962, 751, 1014 spots in HM-1; n=3666, 99, 1042, 3666, 99, 1042 in HM-2; n=3092, 1230, 591, 3092, 1230, 591 in HM-3. The lower whisker, lower hinge, line inside box, upper hinge, and upper whisker represent the minimum, lower quartile, median, upper quartile, and maximum calculated without outlier values which are more than 1.5 * inter-quartile range away from the hinges and are shown in separate dots.

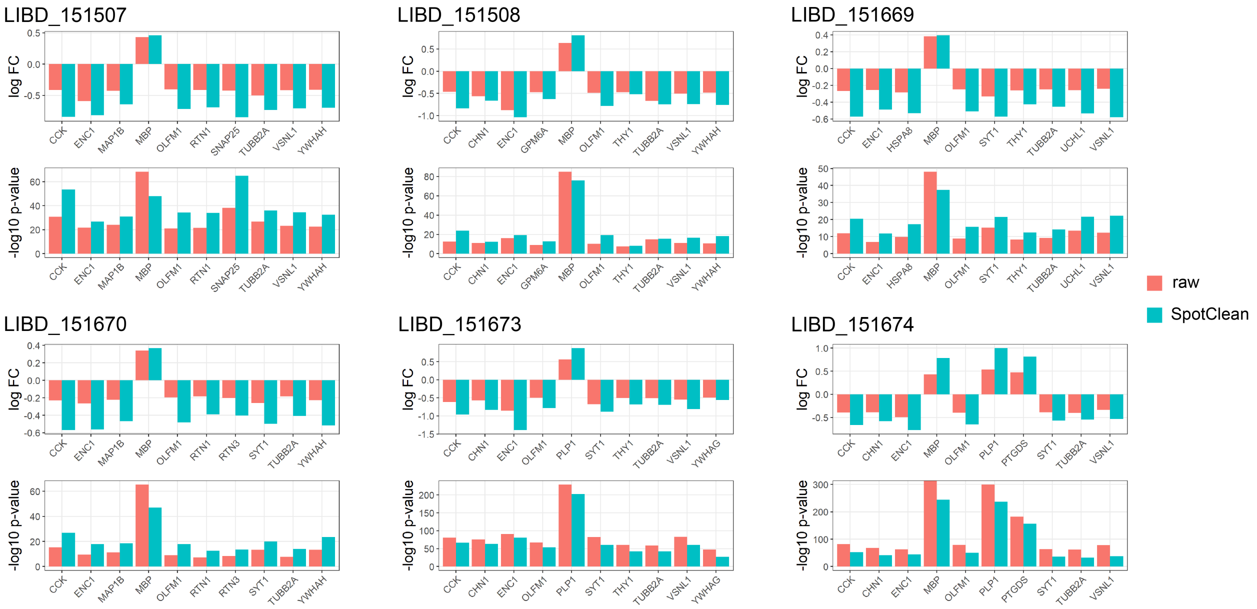

Supplementary Figure 12: Shown for the six human brain sample datasets are the fold changes and p-values for 10 genes known to be differentially expressed (DE) between WM and Layer6 for the raw data (salmon) and SpotClean processed data (turquoise). By reducing noise due to spot-swapped UMIs, SpotClean improves fold changes and p-values for the majority of known DE genes.

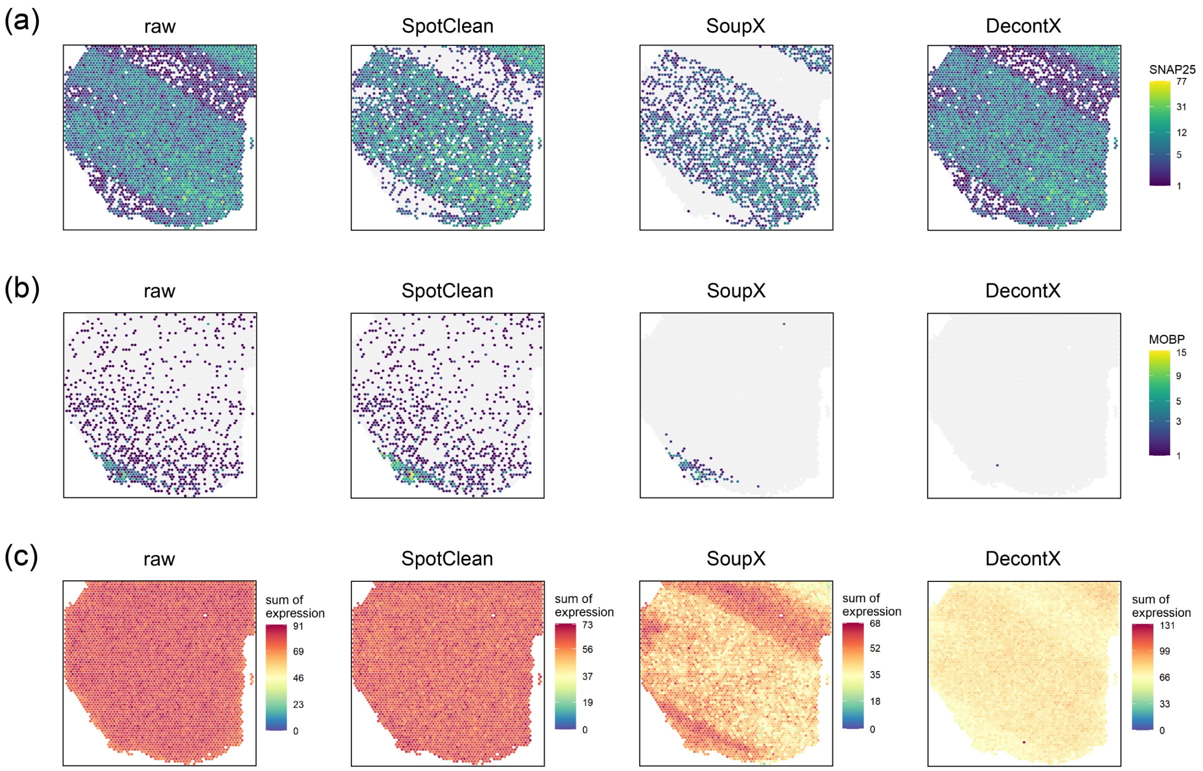

Supplementary Figure 13: Data from the human brain sample LIBD_151507. Data decontaminated by SpotClean (middle left), SoupX (middle right), and DecontX (right) for SNAP25 (a) and MOBP (b). The raw data is shown left. SoupX decontaminates SNAP25, but reduces expression even in Layer2-Layer6, where SNAP25 expression is expected to be high; DecontX imposes little change on this marker's expression. SoupX works well for MOBP, but DecontX removes almost all of the signal for this marker. Panel (c) shows results from SimV data generated using sample LIBD_151507; 500 spatially varying (SV) genes and 500 genes showing no change in expression across the slide were simulated (non-SV). Shown far left in panel (c) is summed expression for the 500 non-SV genes. To ensure that the summation is not dominated by a few highly expressed genes, gene-specific expression was scaled so that the maximum value of each gene equals 1. The same sum is shown for data decontaminated by SpotClean (middle left), SoupX (middle right), and DecontX (right). SoupX and DecontX impose artificial patterns upon non-SV genes.

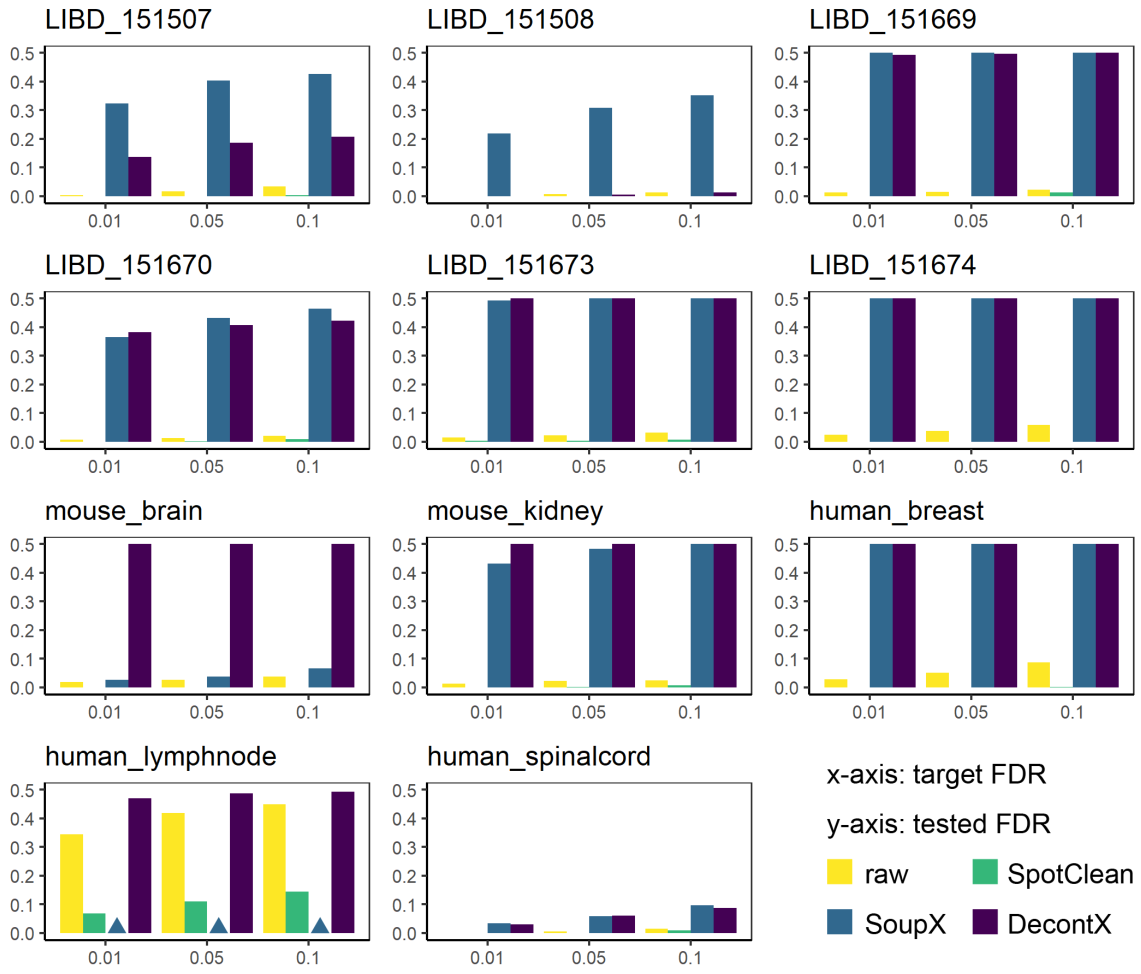

Supplementary Figure 14: SimV simulated data containing spatially varying (SV) genes was simulated for 11 datasets using input from the publicly available dataset indicated. SpatialDE[4] was applied to the simulated data following decontamination by SpotClean, SoupX[5], and DecontX[6]. Shown is the observed false discovery rate (FDR) for the raw data and each dataset following decontamination for three target FDR cut-offs. The upper bound on observed FDR is 0.5 since there are 50% simulated SV genes and 50% simulated non-SV genes. SoupX failed to run on the human_lymphnode data (NA shown as triangles). SoupX and DecontX have increased FDR for many datasets as they impose variability on non-SV genes during decontamination (Supplementary Section S1 and Supplementary Figure 13).

Dataset	Prop. UMIs in Background	Number of background spots	Normalized prop. UMIs in Background	LPSS
LIBD_151507	6.4%	766	0.0084%	NA
LIBD_151508	4.2%	606	0.0069%	NA
LIBD_151669	8.3%	1329	0.0062%	NA
LIBD_151670	7.6%	1494	0.0051%	NA
LIBD_151673	8.6%	1353	0.0064%	NA
LIBD_151674	10.4%	1319	0.0079%	NA
mouse_brain	8.3%	2290	0.0036%	NA
mouse_kidney	7.7%	3554	0.0022%	NA
human_breast	4.3%	1005	0.0043%	NA
human_lymphnode	10.7%	957	0.0112%	NA
human_spinalcord	14.2%	2180	0.0065%	NA
HM-1	20.8%	2962	0.0070%	14.9%
HM-2	17.6%	3666	0.0048%	10.2%
HM-3	26.9%	3092	0.0087%	13.3%

Supplementary Table 1: The proportion of UMI counts in background spots, the number of background spots, the per-spot proportion of UMI counts in background spots, and the LPSS (which is only defined for the three chimeric datasets). The proportion of UMI counts in background spots serves as an underestimate of the proportion of spot-swapped UMI counts (since the proportion quantifies tissue-to-background swapping but does not account for tissue-to-tissue swapping). The proportion depends on tissue coverage since experiments with relatively few background spots will necessarily have a lower proportion of UMI counts in the background. Consequently, we also report the normalized proportion, which is the proportion of UMI counts in background spots divided by the number of background spots in each dataset. LPSS is defined in the chimeric experiment as the proportion of misclassified reads in tissue spots (mouse reads in human spots and human reads in mouse spots). This is a lower bound as it does not account for spot swapping within species (e.g. reads from mouse spot t bound by probes at mouse spot t'), and it does not count reads in background spots.

Dataset	No decontamination	SpotClean	SoupX	DecontX
LIBD_151507	31.334	**15.041**	NA	83.016
LIBD_151508	26.477	**12.987**	NA	53.596
LIBD_151669	21.931	**11.745**	NA	267.124
LIBD_151670	17.903	**10.304**	NA	70.851
LIBD_151673	22.121	**11.682**	NA	56.747
LIBD_151674	25.861	**13.361**	108.916	57.304
mouse_brain	24.979	**9.896**	779.811	278.374
mouse_kidney	12.114	**7.810**	291.890	119.825
human_breast	14.278	**9.605**	139.924	72.129
human_lymphnode	113.216	**30.261**	486.128	189.395
human_spinalcord	126.197	**13.191**	163.898	187.928

Supplementary Table 2: Average mean squared error (MSE) between true and decontaminated gene expression (average taken over 3000 genes) in 11 SimI datasets simulated using input from the dataset indicated. NA denotes datasets for which the corresponding method failed to run. The lowest MSE for each dataset is bolded.

Dataset	No decontamination	SpotClean	SoupX	DecontX
LIBD_151507	30.720	**14.957**	NA	58.651
LIBD_151508	26.102	**12.909**	NA	122.332
LIBD_151669	21.610	**12.001**	NA	266.682
LIBD_151670	17.452	**10.221**	NA	154.391
LIBD_151673	21.472	**12.172**	NA	74.319
LIBD_151674	25.131	**13.469**	NA	57.744
mouse_brain	24.161	**9.625**	824.134	284.147
mouse_kidney	12.165	**7.903**	319.903	121.810
human_breast	13.790	**9.987**	118.043	71.458
human_lymphnode	108.288	**31.581**	464.735	196.503
human_spinalcord	122.037	**14.431**	181.217	515.027

Supplementary Table 3: Average mean squared error (MSE) between true and decontaminated gene expression (average taken over 3000 genes) in 11 SimII datasets simulated using input from the dataset indicated. NA denotes datasets for which the corresponding method failed to run. The lowest MSE for each dataset is bolded.

Dataset	No decontamination	SpotClean	SoupX	DecontX
LIBD_151507	32.472	**14.998**	NA	87.570
LIBD_151508	27.371	**13.248**	NA	196.679
LIBD_151669	22.892	**11.710**	NA	81.707
LIBD_151670	18.538	**10.255**	62.989	63.855
LIBD_151673	23.502	**11.719**	NA	184.707
LIBD_151674	27.832	**13.372**	NA	61.567
mouse_brain	26.856	**9.508**	685.702	284.959
mouse_kidney	12.989	**7.912**	302.584	119.542
human_breast	15.222	**9.953**	135.952	74.705
human_lymphnode	120.495	**28.026**	534.534	195.524
human_spinalcord	133.396	**13.414**	186.904	563.552

Supplementary Table 4: Average mean squared error (MSE) between true and decontaminated gene expression (average taken over 3000 genes) in 11 SimIII datasets simulated using input from the dataset indicated. NA denotes datasets for which the corresponding method failed to run. The lowest MSE for each dataset is bolded.

Dataset	No decontamination	SpotClean	SoupX	DecontX
LIBD_151507	36.056	**15.388**	NA	144.595
LIBD_151508	30.580	**13.639**	NA	141.042
LIBD_151669	25.767	**12.677**	NA	79.926
LIBD_151670	21.365	**10.553**	NA	196.039
LIBD_151673	27.271	**12.769**	97.401	339.906
LIBD_151674	32.751	**13.967**	NA	67.959
mouse_brain	31.022	**9.419**	829.479	458.233
mouse_kidney	14.756	**7.771**	331.323	131.421
human_breast	16.731	**9.348**	136.516	76.562
human_lymphnode	132.168	**29.893**	523.708	209.594
human_spinalcord	152.092	**13.154**	223.067	215.555

Supplementary Table 5: Average mean squared error (MSE) between true and decontaminated gene expression (average taken over 3000 genes) in 11 SimIV datasets simulated using input from the dataset indicated. NA denotes datasets for which the corresponding method failed to run. The lowest MSE for each dataset is bolded.

Dataset	Link
six human brain samples	https://github.com/LieberInstitute/spatialLIBD
mouse_brain	https://support.10xgenomics.com/spatial-gene-expression/datasets/1.1.0/V1_Adult_Mouse_Brain
mouse_kidney	https://support.10xgenomics.com/spatial-gene-expression/datasets/1.1.0/V1_Mouse_Kidney
human_breast	https://support.10xgenomics.com/spatial-gene-expression/datasets/1.1.0/V1_Breast_Cancer_Block_A_Section_2
human_breast_2	https://support.10xgenomics.com/spatial-gene-expression/datasets/1.3.0/Visium_FFPE_Human_Breast_Cancer
human_lymphnode	https://support.10xgenomics.com/spatial-gene-expression/datasets/1.1.0/V1_Human_Lymph_Node
human_spinalcord	https://support.10xgenomics.com/spatial-gene-expression/datasets/1.2.0/Targeted_Visium_Human_SpinalCord_Neuroscience
human_colorectal	https://www.10xgenomics.com/resources/datasets/human-colorectal-cancer-whole-transcriptome-analysis-1-standard-1-2-0
human_pancreatic	https://www.ncbi.nlm.nih.gov/geo/query/acc.cgi?acc=GSE111672
Two datasets from Slide-seqV2	https://singlecell.broadinstitute.org/single_cell/study/SCP815/highly-sensitive-spatial-transcriptomics-at-near-cellular-resolution-with-slide-seqv2#study-download
GSE169749	https://www.ncbi.nlm.nih.gov/geo/query/acc.cgi?acc=GSE169749
GSE178361	https://www.ncbi.nlm.nih.gov/geo/query/acc.cgi?acc=GSE178361
GSE188888	https://www.ncbi.nlm.nih.gov/geo/query/acc.cgi?acc=GSE188888
GSE190595	https://www.ncbi.nlm.nih.gov/geo/query/acc.cgi?acc=GSE190595
GSE193460	https://www.ncbi.nlm.nih.gov/geo/query/acc.cgi?acc=GSE193460

Supplementary Table 6: Links to 30 publicly available spatial transcriptomics datasets (27 from the 10x Visium, 1 from the SpatialTranscriptomics, and 2 from the Slide-seqV2 protocol).

Gene	-log10_pval	logfc	t	CI	df
ENC1	21.6772	-0.59211	-10.0715	-0.77218,-0.52024	716.6266
TUBB2A	26.82923	-0.5005	-11.4299	-0.87942,-0.62153	611.6922
MBP	68.23467	0.43074	20.62996	1.31259,1.58892	502.7044
MAP1B	24.09136	-0.4268	-10.7987	-0.90317,-0.62517	558.5923
SNAP25	38.24431	-0.42293	-14.1973	-1.12206,-0.84929	526.9102
VSNL1	23.18776	-0.41835	-10.5489	-0.85222,-0.58468	578.5369
CCK	30.67843	-0.41324	-12.3489	-0.97287,-0.70589	605.6358
RTN1	21.56528	-0.41252	-10.0983	-0.77982,-0.5259	616.3649
YWHAH	22.66223	-0.40974	-10.4066	-0.85579,-0.58405	586.1957
OLFM1	21.10679	-0.4048	-9.99412	-0.8003,-0.53742	590.4734

Supplementary Table 7: Summary statistics of t-test results for known DE genes in Figure 3b in LIBD_151507 raw data. -log10_pval: $-\log_{10}$ transformed p-value. logfc: log transformed fold change. t: t statistic. CI: 95% confidence interval. df: degrees of freedom.

Gene	-log10_pval	logfc	t	CI	df
ENC1	26.81212	-0.81797	-11.2768	-0.84106,-0.59168	828.6978
TUBB2A	36.07802	-0.73195	-13.3537	-1.07278,-0.79781	785.5271
MBP	47.97652	0.458097	16.37517	1.41258,1.79774	507.8979
MAP1B	30.97989	-0.64203	-12.3232	-1.15413,-0.83691	696.4673
SNAP25	64.89745	-0.85097	-19.0397	-1.70572,-1.38683	710.7584
VSNL1	34.64576	-0.70785	-13.0801	-1.12332,-0.83014	751.8196
CCK	53.54393	-0.84157	-16.7963	-1.49164,-1.17946	779.5883
RTN1	34.02306	-0.69379	-12.915	-1.0405,-0.76594	787.7664
YWHAH	32.53661	-0.6964	-12.6217	-1.1375,-0.83128	752.0688
OLFM1	34.35643	-0.71864	-13.0144	-1.08758,-0.80248	755.607

Supplementary Table 8: Summary statistics of t-test results for known DE genes in Figure 3b in LIBD_151507 data decontaminated by SpotClean. -log10_pval: $-\log_{10}$ transformed p-value. logfc: log transformed fold change. t: t statistic. CI: 95% confidence interval. df: degrees of freedom.

Supplementary Section S1: Sources of contamination in RNA-sequencing experiments

We define UMI counts for a given gene in a given sample as contaminated if at least one of the counts did not arise from that gene in that sample. Common sources of contamination include barcode swapping (also called index hopping) and contamination by ambient RNA. Barcode swapping refers to a barcode from one sample binding reads from another once samples are pooled together for sequencing. Specifically, most RNA-sequencing protocols attach barcodes (sample-specific strings of nucleotides) to mRNA transcripts in a sample prior to pooling samples for sequencing so that, following sequencing, sample-specific transcript abundance can be estimated. This is the case for the 10x Visium spatial transcriptomics protocol, where mRNA binds barcodes that are spatially addressed to a given tissue spot, then are released and pooled for sequencing. The process is not without error, and numerous studies have found evidence of barcode swapping, where a barcode specific to one sample binds reads from another at random[7–10]. This is distinct from the spot swapping artifact detailed here since spot swapping is not at random. Rather, with spot swapping, the probability of a spot-specific barcode binding reads from another spot increases as the distance between spots decreases. As the statistical methods developed to adjust for barcode swapping[10] do not accommodate the spatial dependence inherent in spot swapping, they are not sufficient in this setting.

A second type of contamination is specific to scRNA-seq experiments. In droplet based scRNA-seq, for example, each droplet ideally contains one cell, and barcodes specific to that droplet bind mRNA from the cell. In practice, however, ambient (cell free) RNA may also bind barcodes from a droplet. As with barcode swapping, robust statistical methods are in place to adjust[5,6], but they are not appropriate for spatial data as they do not accommodate the spatial dependence. Supplementary Figure 13 shows results from two popular decontamination methods, SoupX[5] and DecontX[6]. Each of these methods begins by clustering single-cell data[6], or taking as input clustering information[5]; decontamination is then performed within cluster. For some marker genes that distinguish between clusters, these methods work well, or at least do not change the data very much. However, in many cases, decontamination reduces signal substantially (Supplementary Figure 13, panels a and b). In addition, since both methods decontaminate all genes within a cluster simultaneously, artificial patterns are imposed upon genes showing no spatial changes (Supplementary Figure 13, panel c). This leads to poor estimates of expression

(Supplementary Tables 2-5) and increased false discoveries in downstream analyses (Supplementary Figure 14). These results should not be taken as evidence that SoupX and DecontX perform poorly in general. That is not the case. Rather, it should be stressed that neither method was designed for spatial data and, consequently, it should not be surprising that they are not sufficient in this setting.

Supplementary Section S2: **Derivation of gradients for estimating contamination parameters.**

Recall that we would like to minimize

$$\left(\widehat{r_\beta}, \widehat{r_\gamma}, \hat{\sigma}, \{\widehat{\mu_{\cdot t}}\}_{t\in I_t}\right) = \underset{r_\beta, r_\gamma, \sigma, \{\mu_{\cdot t}\}_{t\in I_t}}{\operatorname{argmin}} \sum_{j\in I_t \cup I_b} \left(X_{\cdot j} - \eta_{\cdot j}\right)^2 \tag{1}$$

where

$$\eta_{\cdot j} = E\left(X_{\cdot j}\right) = \begin{cases} \sum_{t\in I_t} \mu_{\cdot t}\, r_\beta \left[r_\gamma \frac{1}{K} + \left(1 - r_\gamma\right) w_{t,j}\right] & \text{, if } j \in I_b \\ \mu_{\cdot j}\left(1 - r_\beta\right) + \sum_{t\in I_t} \mu_{\cdot t}\, r_\beta \left[r_\gamma \frac{1}{K} + \left(1 - r_\gamma\right) w_{t,j}\right] & \text{, if } j \in I_t \end{cases} \tag{2}$$

Rewriting the problem in matrix representation, let $\mu = \left(\mu_{\cdot 1}, \dots, \mu_{\cdot K_t}\right)^T$ and $X = \left(\{X_{\cdot j}\}_{j\in I_b}, \{X_{\cdot j}\}_{j\in I_t}\right)^T$. Denote the proximal contamination weight matrix $W = \begin{pmatrix} W_1 \\ W_2 \end{pmatrix}$, where W_1 is a $K_b \times K_t$ matrix containing the Gaussian weights between background and tissue spots, and W_2 is a $K_t \times K_t$ matrix containing the Gaussian weights between pairs of tissue spots. The column sums of W are equal to 1 based on its definition. Let I_{K_t} be the identity matrix with dimension K_t. Let J_1 and J_2 be $K_b \times K_t$ and $K_t \times K_t$ matrices of ones representing the distal contamination weights. The residual sum of squares (RSS) becomes

$$RSS = \left\| X - \left[\left(1 - r_\beta\right)\begin{pmatrix} 0 \\ I_{K_t} \end{pmatrix} + r_\beta\left(1 - r_\gamma\right)\begin{pmatrix} W_1 \\ W_2 \end{pmatrix} + \frac{r_\beta r_\gamma}{K}\begin{pmatrix} J_1 \\ J_2 \end{pmatrix}\right]\mu \right\|_{L_2}^2 \tag{3}$$

The gradients of unknown parameters are then calculated as

$$\begin{aligned} \frac{\partial \mathrm{RSS}}{\partial \mu} = 2\Bigg[&\left(1 - r_\beta\right)^2 I_{K_t} + {r_\beta}^2\left(1 - r_\gamma\right)^2 W^T W + r_\beta\left(1 - r_\beta\right)\left(1 - r_\gamma\right)\left(W_2^T + W_2\right) \\ &+ \frac{r_\beta r_\gamma\left(2 - r_\beta r_\gamma\right)}{K} J_2\Bigg]\mu \\ &- 2\left[\left(1 - r_\beta\right)\begin{pmatrix} 0 & I_{K_t} \end{pmatrix} X + r_\beta\left(1 - r_\gamma\right) W^T X + \frac{r_\beta r_\gamma}{K}\begin{pmatrix} J_1 \\ J_2 \end{pmatrix}^T X\right] \end{aligned} \tag{4}$$

$$\begin{aligned}\frac{\partial RSS}{\partial r_\beta} = \mu^T \Bigg[& 2(r_\beta - 1) I_{K_t} + 2 r_\beta (1 - r_\gamma)^2 W^T W \\ & + (1 - 2 r_\beta)(1 - r_\gamma)(W_2^T + W_2) + \frac{2 r_\gamma - 2 r_\beta {r_\gamma}^2}{K} J_2 \Bigg] \mu \\ & - 2 X^T \left[(1 - r_\gamma) W + \frac{r_\gamma}{K} \begin{pmatrix} J_1 \\ J_2 \end{pmatrix} - \begin{pmatrix} 0 \\ I_{K_t} \end{pmatrix} \right] \mu \end{aligned} \tag{5}$$

$$\begin{aligned}\frac{\partial RSS}{\partial r_\gamma} = \mu^T & \left[2 {r_\beta}^2 (r_\gamma - 1) W^T W + r_\beta (r_\beta - 1)(W_2^T + W_2) + \frac{2 r_\beta - 2 {r_\beta}^2 r_\gamma}{K} J_2 \right] \mu \\ & - 2 X^T \left[\frac{r_\beta}{K} \begin{pmatrix} J_1 \\ J_2 \end{pmatrix} - r_\beta W \right] \mu \end{aligned} \tag{6}$$

Supplementary Section S3: Derivation of the EM algorithm for estimating true gene expression values.

Recall that the observed data $\mathcal{D} = \{X_{g,j}\}_{g\in G, j\in I_t \cup I_b}$ has log-likelihood

$$l_{\mathcal{D}} = \sum_{g\in G} \sum_{j\in I_t\cup I_b} l_{X_{g,j}} = \sum_{g\in G} \sum_{j\in I_t\cup I_b} \{X_{g,j} \log \eta_{g,j} - \eta_{g,j}\} + \text{constant} \tag{7}$$

and the complete data are $\mathcal{C} = \{S_{g,t}, B_{g,t,j}\}_{g\in G, t\in I_t, j\in I_t\cup I_b}$ with log-likelihood

$$l_{\mathcal{C}} = \sum_{g\in G} \sum_{t\in I_t} l_{S_{g,t}} + \sum_{g\in G} \sum_{t\in I_t} \sum_{j\in I_t\cup I_b} l_{B_{g,t,j}} \tag{8}$$

$$\begin{aligned} &= \sum_{g\in G} \sum_{t\in I_t} \left[S_{g,t} \log\left(\mu_{g,t}(1-r_\beta)\right) - \mu_{g,t}(1-r_\beta)\right] \\ &+ \sum_{g\in G} \sum_{t\in I_t} \sum_{j\in I_t\cup I_b} \left[B_{g,t,j} \log\left(\mu_{g,t} r_\beta \left[(1-r_\gamma) w_{t,j} + r_\gamma \frac{1}{K}\right]\right)\right. \\ &\left. - \mu_{g,t} r_\beta \left[(1-r_\gamma) w_{t,j} + r_\gamma \frac{1}{K}\right]\right] + \text{constant} \end{aligned} \tag{9}$$

$$\begin{aligned} &= \sum_{g\in G} \sum_{t\in I_t} \left(S_{g,t} \log\left(\mu_{g,t}(1-r_\beta)\right)\right. \\ &\left. + \sum_{j\in I_t\cup I_b} \left[B_{g,t,j} \log\left(\mu_{g,t} r_\beta \left[(1-r_\gamma) w_{t,j} + r_\gamma \frac{1}{K}\right]\right)\right] - \mu_{g,t} \right) \\ &+ \text{constant} \end{aligned} \tag{10}$$

Let $\{\mu_{g,t}^{(n)}\}_{g\in G, t\in I_t}$ be the parameter values at the n-th iteration. The E-step involves computation of the expectation of latent variables conditioning on observed data and parameter values at the current iteration. Given the fact that if $U \sim \text{Poisson}(a)$, $V \sim \text{Poisson}(b)$, and U and V are independent, then $U|(U+V) \sim \text{Binomial}(U+V, \frac{a}{a+b})$, and we have

$$S_{g,t}^{(n)} \coloneqq E[S_{g,t}|\mathcal{D}] = E[S_{g,t}|X_{g,t}] = X_{g,t} \frac{\mu_{g,t}^{(n)}(1-r_\beta)}{\eta_{g,t}^{(n)}} \tag{11}$$

$$B_{g,t,j}^{(n)} \coloneqq E\left[B_{g,t,j}\middle|\mathcal{D}\right] = E\left[B_{g,t,j}\middle|X_{g,j}\right] = X_{g,j}\frac{\mu_{g,t}^{(n)} r_\beta\left[(1-r_\gamma)w_{t,j} + r_\gamma\frac{1}{K}\right]}{\eta_{g,j}^{(n)}} \tag{12}$$

The M-step involves maximizing the complete log-likelihood after plugging in the conditional expectations in the E-step:

$$\begin{aligned} l_{\mathcal{C}}^{(n)} = \sum_{g\in G}\sum_{t\in I_t}\Bigg(& S_{g,t}^{(n)}\log\left(\mu_{g,t}(1-r_\beta)\right) \\ & + \sum_{j\in I_t\cup I_b} B_{g,t,j}^{(n)}\log\left(\mu_{g,t} r_\beta\left[(1-r_\gamma)w_{t,j} + r_\gamma\frac{1}{K}\right]\right) - \mu_{g,t}\Bigg) \end{aligned} \tag{13}$$

$$\frac{\partial l_{\mathcal{C}}^{(n)}}{\partial \mu_{g,t}} = \frac{S_{g,t}^{(n)} + \sum_{j\in I_t\cup I_b} B_{g,t,j}^{(n)}}{\mu_{g,t}} - 1 \tag{14}$$

The M-step becomes

$$\mu_{g,t}^{(n+1)} = S_{g,t}^{(n)} + \sum_{j\in I_t\cup I_b} B_{g,t,j}^{(n)} \tag{15}$$

References

1. Ståhl, P. L. *et al.* Visualization and analysis of gene expression in tissue sections by spatial transcriptomics. *Science* vol. 353 78–82 (2016).
2. Maynard, K. R. *et al.* Transcriptome-scale spatial gene expression in the human dorsolateral prefrontal cortex. *Nature Neuroscience* **24**, 425–436 (2021).
3. Stickels, R. R. *et al.* Highly sensitive spatial transcriptomics at near-cellular resolution with Slide-seqV2. *Nature Biotechnology* **39**, 313–319 (2021).
4. Svensson, V., Teichmann, S. A. & Stegle, O. SpatialDE: Identification of spatially variable genes. *Nature Methods* **15**, 343–346 (2018).
5. Young, M. D. & Behjati, S. SoupX removes ambient RNA contamination from droplet-based single-cell RNA sequencing data. *GigaScience* **9**, 1–10 (2020).
6. Yang, S. *et al.* Decontamination of ambient RNA in single-cell RNA-seq with DecontX. *Genome Biology* **21**, 57 (2020).
7. Costello, M. *et al.* Characterization and remediation of sample index swaps by non-redundant dual indexing on massively parallel sequencing platforms. *BMC Genomics* (2018) doi:10.1186/s12864-018-4703-0.
8. Farouni, R., Djambazian, H., Ferri, L. E., Ragoussis, J. & Najafabadi, H. S. Model-based analysis of sample index hopping reveals its widespread artifacts in multiplexed single-cell RNA-sequencing. *Nature Communications* (2020) doi:10.1038/s41467-020-16522-z.
9. Larsson, A. J. M., Stanley, G., Sinha, R., Weissman, I. L. & Sandberg, R. Computational correction of index switching in multiplexed sequencing libraries. *Nature Methods* (2018) doi:10.1038/nmeth.4666.
10. Griffiths, J. A., Richard, A. C., Bach, K., Lun, A. T. L. & Marioni, J. C. Detection and removal of barcode swapping in single-cell RNA-seq data. *Nature Communications* **9**, 1–6 (2018).
