# Supplementary material for: SpotClean adjusts for spot swapping in spatial transcriptomics data
Source: Nat Commun. 2022 May 27;13:2971. doi: 10.1038/s41467-022-30587-y (PMC9142522; doi:10.1038/s41467-022-30587-y)
Supplement: Supplementary file 3 — Reporting Summary [file 41467_2022_30587_MOESM3_ESM.pdf]

nature research

Corresponding author(s): Christina Kendziorski

Last updated by author(s): May 9, 2022

Reporting Summary

Nature Research wishes to improve the reproducibility of the work that we publish. This form provides structure for consistency and transparency in reporting. For further information on Nature Research policies, see our Editorial Policies and the Editorial Policy Checklist.

Statistics

For all statistical analyses, confirm that the following items are present in the figure legend, table legend, main text, or Methods section.

n/a	Confirmed	
☐	☒	The exact sample size (n) for each experimental group/condition, given as a discrete number and unit of measurement
☐	☒	A statement on whether measurements were taken from distinct samples or whether the same sample was measured repeatedly
☐	☒	The statistical test(s) used AND whether they are one- or two-sided<br>*Only common tests should be described solely by name; describe more complex techniques in the Methods section.*
☐	☒	A description of all covariates tested
☐	☒	A description of any assumptions or corrections, such as tests of normality and adjustment for multiple comparisons
☐	☒	A full description of the statistical parameters including central tendency (e.g. means) or other basic estimates (e.g. regression coefficient) AND variation (e.g. standard deviation) or associated estimates of uncertainty (e.g. confidence intervals)
☐	☒	For null hypothesis testing, the test statistic (e.g. F, t, r) with confidence intervals, effect sizes, degrees of freedom and P value noted<br>*Give P values as exact values whenever suitable.*
☐	☒	For Bayesian analysis, information on the choice of priors and Markov chain Monte Carlo settings
☐	☒	For hierarchical and complex designs, identification of the appropriate level for tests and full reporting of outcomes
☐	☒	Estimates of effect sizes (e.g. Cohen's d, Pearson's r), indicating how they were calculated

Our web collection on statistics for biologists contains articles on many of the points above.

Software and code

Policy information about availability of computer code

Data collection	bcl2fastq v2.20.0.422 was used for basecalling and demultiplexing.<br>Tissues were manually aligned using Loupe Browser v4.2.0.<br>Raw fastq files were aligned and quantified using Space Ranger v1.2.2 under default settings.

Data analysis	The following software and packages were used in the analysis: R-4.0.2; R/SpotClean-0.99.0; R/SoupX-1.5.0; R/celda-1.5.11; R/Seurat-3.2.2; R/scran-1.17.20; R/reticulate-1.16; R/SPOTlight-0.1.7; Python-3.7.4; Python/spatialde-1.1.3; FastQC-0.11.7; MultiQC-1.9; Space Ranger-1.2.2; Loupe Browser-4.2.0.<br>The R package SpotClean is available at https://github.com/zijianni/SpotClean and will be submitted to Bioconductor. Codes for simulation and real data analyses as well as processed data can be found at https://github.com/zijianni/codes_for_SpotClean_paper.<br>SpotClean is a probabilistic model that estimates underlying true gene expression and hyperparameters using L-BFGS-B gradient descent and EM algorithm.<br>Raw sequencing data for the chimeric samples were processed using Space Ranger.<br>The count matrices were normalized using scran.<br>Seurat pipeline was applied to analyze case study datasets for variable gene selection, scaling, dimension reduction, clustering, and UMAP visualization.<br>Differential expression (DE) analyses were conducted using two-sample two-sided t-tests. p-values were adjusted using Benjamini-Hochberg correction.<br>Cell type compositions were estimated using SPOTlight.<br>Spearman correlation was used to evaluate the similarity between spots and single cells.<br>Spatial clustering was conducted using BayesSpace.<br>SpotClean was benchmarked with other single-cell RNA-seq decontamination methods SoupX and DecontX.

For manuscripts utilizing custom algorithms or software that are central to the research but not yet described in published literature, software must be made available to editors and reviewers. We strongly encourage code deposition in a community repository (e.g. GitHub). See the Nature Research guidelines for submitting code & software for further information.

Data

Policy information about availability of data

All manuscripts must include a data availability statement. This statement should provide the following information, where applicable:

- Accession codes, unique identifiers, or web links for publicly available datasets
- A list of figures that have associated raw data
- A description of any restrictions on data availability

Raw sequence data for the 3 human-mouse chimeric experiments are available at GEO (accession number: GSE178221). Links to 16 public spatial transcriptomics datasets are available in Supplementary Table 6. The human breast cancer single-cell RNA-seq data from Chung et al.2313 is available at GEO (accession number: GSE75688). The human colorectal cancer single-cell RNA-seq data from Li et al.2416 is available at GEO (accession number: GSE81861). Additional datasets used to investigate permeabilization times are available at GEO (accession numbers: GSE169749, GSE178361, GSE188888, GSE190595, and GSE193460). Processed data for reproducing results in our studies are available at Zenodo27. The GRCh38+mm10 reference genome is available at 10x Genomics (refdata-gex-GRCh38-and-mm10-2020-A).

Field-specific reporting

Please select the one below that is the best fit for your research. If you are not sure, read the appropriate sections before making your selection.

[x] Life sciences   [ ] Behavioural & social sciences   [ ] Ecological, evolutionary & environmental sciences

For a reference copy of the document with all sections, see nature.com/documents/nr-reporting-summary-flat.pdf

Life sciences study design

All studies must disclose on these points even when the disclosure is negative.

Sample size	No sample-size calculation was performed. Our study identifies and provides an approach to correct for a general technical artifact (spot swapping) in spatial transcriptomics experiments. We generated and then analyzed three chimeric samples to show the existence and quantify the extent of spot swapping in the experiments. Spot swapping was detected in all of our chimeric samples as well as many other publicly available datasets.
Data exclusions	No data were excluded from the analyses.
Replication	We validated the technical artifact in 10x Visium spatial transcriptomics experiments using our in-house samples, and detected the artifact in 16 public datasets across multiple platforms. The effect is ubiquitous. Any existing or new data coming from these protocols can be used to validate our finding following similar analyses.
Randomization	This is not relevant to our study. Our study does not involve any comparison of experimental groups under different treatments.
Blinding	Blinding was not relevant to our study. Our study does not involve any comparison of experimental groups under different treatments.

Reporting for specific materials, systems and methods

We require information from authors about some types of materials, experimental systems and methods used in many studies. Here, indicate whether each material, system or method listed is relevant to your study. If you are not sure if a list item applies to your research, read the appropriate section before selecting a response.

Materials & experimental systems

n/a	Involved in the study	
☒	☐	Antibodies
☒	☐	Eukaryotic cell lines
☒	☐	Palaeontology and archaeology
☐	☒	Animals and other organisms
☐	☒	Human research participants
☒	☐	Clinical data
☒	☐	Dual use research of concern

Methods

n/a	Involved in the study	
☒	☐	ChIP-seq
☒	☐	Flow cytometry
☒	☐	MRI-based neuroimaging

Animals and other organisms

Policy information about studies involving animals; ARRIVE guidelines recommended for reporting animal research

Laboratory animals	Mus musculus, C57BL/6J, male, 10 weeks old
Wild animals	The study did not involve wild animals.
Field-collected samples	The study did not involve samples collected from the field.
Ethics oversight	All mouse husbandry and experimental procedures were performed in accordance and compliance with policies approved by the University of Wisconsin Research Animals Research and Compliance committee.

Note that full information on the approval of the study protocol must also be provided in the manuscript.

Human research participants

Policy information about studies involving human research participants

Population characteristics	The covariate-relevant population characteristics are as follows: two patients at the ages of 60 and 39 with current diagnoses of non-melanoma skin cancer requiring Dermatologic surgery for treatment.
Recruitment	The two participants in this study were randomly recruited in clinic on a single day during routine Dermatologic surgery. No other potential participants were approached because additional tissue wasn't needed. They consented to have their normal residual tissue from Dermatologic surgery used for this study. While this recruitment is biased toward patients who needed a Dermatologic procedure, given that we intentionally sampled normal skin from these patients, we do not anticipate an impact on our results.
Ethics oversight	Fresh sections of normal human skin tissue were obtained with informed consent during routine dermatologic surgery under protocols approved by the University of Wisconsin School of Medicine and Public Health Institutional Review Board.

Note that full information on the approval of the study protocol must also be provided in the manuscript.
